# Alarm Pheromone Responses Depend on Genotype, but Not on the Presence of Facultative Endosymbionts in the Pea Aphid *Acyrthosiphon pisum*

**DOI:** 10.3390/insects12010043

**Published:** 2021-01-08

**Authors:** Cesar Auguste Badji, Zoé Sol-Mochkovitch, Charlotte Fallais, Corentin Sochard, Jean-Christophe Simon, Yannick Outreman, Sylvia Anton

**Affiliations:** 1 IGEPP, INRAE, Institut Agro, University Rennes, CEDEX, 49045 Angers, France; cesar.badji@ufape.edu.br (C.A.B.); zoe-sol@hotmail.fr (Z.S.-M.); charlotte.fallais@gmail.com (C.F.); 2 IGEPP, INRAE, Institut Agro, University Rennes, CEDEX, 35000 Rennes, France; corentin.sochard@etudiant.univ-rennes1.fr (C.S.); yannick.outreman@agrocampus-ouest.fr (Y.O.); 3 IGEPP, INRAE, Institut Agro, University Rennes, CEDEX, 35653 Le Rheu, France; jean-christophe.simon@inrae.fr

**Keywords:** Hemiptera, endosymbiotic bacteria, *Hamiltonella defensa*, aphid alarm pheromone, avoidance behaviour, olfactometry, electroantennography

## Abstract

**Simple Summary:**

Aphids are severe pests on many plants, and understanding their olfactory-guided behaviour is essential for the development of alternative management strategies. The pea aphid, Acyrthosiphon pisum, is a good model to understand the influence of different factors on olfactory-guided behaviour, because it encompasses several biotypes, each specialised on different legumes, and harbours different facultative symbionts that influence the insect hosts in many ways. We investigate here whether the aphid genotype and facultative symbionts influence behavioural and antennal responses to the alarm pheromone E-β farnesene in pea aphids. This alarm pheromone is released in case of danger and elicits escape behaviour of conspecifics. For that, we used different pea aphid clones specialised on different host plants and derived lines that harbour or not facultative endosymbiotic bacteria. In behavioural assays, we found that, indeed, aphid genotypes respond differentially to E-β farnesene, whereas the presence of an endosymbiont conferring protection against natural enemies does not modify responses to the alarm pheromone. Electrophysiological recordings from the olfactory organ, the antenna, revealed significant differences in sensitivity between aphid genotypes but not as a function of endosymbionts, corresponding to the behavioural results.

**Abstract:**

Aphids use an alarm pheromone, E-β farnesene (EBF), to warn conspecifics of potential danger. The antennal sensitivity and behavioural escape responses to EBF can be influenced by different factors. In the pea aphid, *Acyrthosiphon pisum,* different biotypes are adapted to different legume species, and within each biotype, different genotypes exist, which can carry or not *Hamiltonella defensa*, a bacterial symbiont that can confer protection against natural enemies. We investigate here the influence of the aphid genotype and symbiotic status on the escape behaviour using a four-way olfactometer and antennal sensitivity for EBF using electroantennograms (EAGs). Whereas the investigated three genotypes from two biotypes showed significantly different escape and locomotor behaviours in the presence of certain EBF doses, the infection with *H. defensa* did not significantly modify the escape behaviour and only marginally influenced the locomotor behaviour at high doses of EBF. Dose-response curves of EAG amplitudes after stimulation with EBF differed significantly between aphid genotypes in correlation with behavioural differences, whereas antennal sensitivity to EBF did not change significantly as a function of the symbiotic status. The protective symbiont *H. defensa* does thus not modify the olfactory sensitivity to the alarm pheromone. How EBF sensitivity is modified between genotypes or biotypes remains to be investigated.

## 1. Introduction

Aphids secrete an alarm pheromone, E-β farnesene (EBF), emitted at the cornicles and common to many species, to warn conspecifics of potential danger, such as, for example, the approach of natural enemies [1]. Aphids exposed to EBF respond with escape (i.e., dropping off the plant) or defensive behaviours, such as kicking [2,3], which has attracted the interest of chemical ecologists, trying to develop alternative plant protection strategies using semiochemicals [4]. However, aphid species and populations are not exposed to the same predation risks, which may select for different responses to EBF [2]. In addition, many aphids host facultative bacterial symbionts that confer a protection against natural enemies [5] but which may alter the responses to EBF as a side effect of the symbiont infection (i.e., cost of carrying the symbiont) or as a way to reduce unnecessary costly defences (e.g., defensive behaviours and alarm pheromone emission).

The pea aphid, *Acyrthosiphon pisum* Harris (Hemiptera, Aphididae), is an excellent model to study the factors that may cause variations of responses to EBF for several reasons. Firstly, the sensory mechanisms underlying EBF detection have been investigated. They detect EBF with receptor neurons situated in large placoid sensilla on the sixth antennal segment, expressing the odorant receptor ApisOR5 [6]. Secondly, at least 15 different biotypes, specialised to different host plants, have been well-characterised in *A. pisum*, and for each biotype, different genotypes are available [7,8,9]. Thirdly, the pea aphid shows a diversity in endosymbiotic bacteria, which can be eliminated or transfected in different genetic backgrounds, and the influence of such bacteria on behavioural traits has been previously reported [10,11,12,13,14]. Finally, like other aphid species, the pea aphid reproduces parthenogenetically during most of its annual life cycle, and thus, clonal individuals can be obtained for experiments, which facilitates the dissection of genetic and environmental components of phenotypic variation.

The strength of behavioural responses upon EBF exposure, by measuring antennal movements, stylet withdrawal, kicking, and leaving the host plant, has previously been shown to vary between certain biotypes of the pea aphid, which are adapted to different host plants. Biotypes adapted to *Medicago sativa* and *Trifolium pratense* responded more strongly than biotypes adapted to *Pisum sativum* [2]. On the other hand, dropping responses upon EBF exposure did not differ significantly between *M. sativa*, *T. pratense*, *Genista tinctoria*, and *G. sagittalis* biotypes [15]. Variations in EBF responses as a function of the genotype and symbiotic status have not been investigated so far.

Microbial symbionts are widespread in the animal kingdom and represent hidden players in many ecological and evolutionary processes [16]. They have been shown to influence many life traits of their host in a wide variety of organisms [17,18], such as nutrition [19], protection against natural enemies [20], or reproduction [21]. However, very little is known on the possible role of endosymbionts on animal sensory systems, such as olfaction. Insect olfactory systems have been shown to be highly plastic, and both internal and external factors modulate the sensitivity to odours crucial for survival and reproduction [22]. Behavioural olfactory plasticity in insects can originate from sensitivity changes in the peripheral (antennal) or central olfactory systems, as shown through electrophysiological and optical imaging investigations [23]. Endosymbionts are another factor to be considered in contributing to the plasticity of sensory systems. Germfree mice, for example, have modified preferences to odorants through changes in the olfactory epithelium [24]. In *Drosophila*, the gut microbiota can modify olfactory-guided microbial preferences and foraging behaviour [25]. In agricultural pest insects, the impact of endosymbionts on the adaptation to a suitable habitat (i.e., host plants and natural enemies), largely dependent on the behaviour in response to volatile signals, has hardly been studied so far.

Eight species of heritable facultative endosymbionts have been reported in the pea aphid, and the various *A. pisum* biotypes generally differ in their respective composition in facultative endosymbionts [26]. For example, the symbiont *Regiella insecticola* is prevalent in the biotype of *A. pisum* specialised on clover, while *Hamiltonella defensa* is dominant in the biotype adapted to alfalfa and fixed in the one specialised on *G. tinctoria* (dyer’s broom) [27,28,29]. As *H. defensa* was shown to confer protection against natural enemies [5], this endosymbiont might modify responses to the alarm pheromone. Indeed, it was shown that defensive behaviours (aggressivity and escape) in an aphid colony attacked by a natural enemy (whether a parasitoid or a predator) were less important when they are infected by *H. defensa* [10,11,13]. This observation leads to the question of whether the reduction of aggressivity and escape in infected aphids might originate from a higher antennal detection threshold of the alarm pheromone and a decrease of the behavioural avoidance response.

We studied here if the avoidance behaviour and antennal detection of EBF vary between three genotypes of *A. pisum* deprived of secondary symbionts, one genotype specialised on *G. tinctoria*, and two genotypes of the biotype specialised on *M. sativa*. We then investigated if the infection with *H. defensa* modifies the avoidance and antennal detection of EBF in lines of the same genotypes but with or without *H. defensa*. We also tested the possible transfer of potential symbiotic effects by injecting different strains of *H. defensa* into a common genotype (*G. tinctoria* biotype). Olfactometer bioassays were used to test the repulsive effect of four doses of EBF on *A. pisum* larvae. We measured the perception by the repulsion score and the locomotor response to the perception by counting the number of transitions between the four zones of the olfactometer. Using electroantennogram recordings (EAGs), antennal sensitivity was evaluated in the three aphid genotypes without a secondary symbiont and in a line with *H. defensa*, which showed slight behavioural differences in EBF responses in the olfactometer assays.

## 2. Materials and Methods

### 2.1. Insects and Endosymbiont Treatments

We used two genotypes specialised on *M. sativa* and one genotype specialised on *G. tinctoria* of the pea aphid, *A. pisum*, which was treated with antibiotics to obtain lines without facultative endosymbionts, and then, new lines were created by reinjecting *H. defensa* originating from the same or a different aphid host [30]. Briefly, first or third instar larvae of the “receiver” genotypes were injected with the haemolymph of a selected line containing the corresponding *H. defensa* (see [30] for more details on the procedure). A total of eight lines deriving from the three genetically distinct clones were used in this study to compare potential intra- and intergenotype differences (Table 1). These lines were chosen following experiments showing evidence for differences in defensive behaviours against parasitoids between clones carrying or not carrying *H. defensa* [13]. Injections with *H. defensa* originating from different genotypes into a common aphid host genotype were done to disentangle potential host and endosymbiont effects on EBF responses. For the sake of simplicity, aphid lines deprived of secondary symbionts are referred hereafter to as “aposymbiotic lines” (although they all still harbour the primary symbiont, *Buchnera aphidicola*).

Aphid lines were reared on faba bean (*Vicia faba*) shoots, the general host plant for all pea aphid biotypes [7,31]. Low densities of aphids were maintained to avoid the formation of winged morphs by renewing host plants twice a week with a maximum of five adult females/plant. Fourth instar larvae (L4) were used in all experiments, because they are easy to recognise and manipulate and respond better than adults to the alarm pheromone [2]. Genotypic identity and the symbiotic status of the experimental lines were checked once every two months by molecular analysis (for details, see [9]). 

### 2.2. Stimuli

E-beta-farnesene (Bedoukian, Danbury, CT, USA) was diluted in hexane in decadic steps from 0.1 ng/μL to 10 μg/μL. Ten microlitres of a solution was applied on a filter paper inside a Pasteur pipette. For olfactometer experiments, four amounts from 0.01 μg to 10 μg of EBF on the filter paper were used. These amounts were shown in preliminary experiments to elicit dose-dependent repulsive responses in various pea aphid clones (data not shown). For electroantennogram (EAG) recordings, a dose-response curve was obtained with doses from 1 ng to 100 μg on the filter paper. In EAG recordings, we also stimulated the antenna with 1-mg cis-3-hexenyl acetate (Sigma-Aldrich, Saint Qentin Fallavier, France), a major component of pea odour [32]. As controls, Pasteur pipettes containing a filter paper with 10 μL of the solvent hexane were used. 

### 2.3. Behavioural Tests

The studied L4 larvae of all eight aphid lines were starved for 1 h before the beginning of the experimentation. A 4-way olfactometer [33] was positioned in a white wooden case. The experiments were done in an environment between 22 and 27 °C, in full artificial light from above by two fluorescent white 18-W cold light tubes during the afternoon, from 1 p.m. to 4:30 p.m. Since movements in the olfactometer can only occur on a horizontal plane, we assumed that light conditions would not bias the aphid behaviour (see, also, the control experiments below). Atmospheric pressure was between 979 Pa and 1015 Pa and the humidity between 27% and 44% in the experimental room. Stimulation pipettes were placed diagonally in the 4-way olfactometer to favour transitions between olfactometer zones: 2 EBF pipettes were positioned diagonally, and hexane pipettes were introduced into the remaining two branches (Figure S1). For control treatments, hexane pipettes were introduced in all four branches. The aphid was then placed in an opening in the centre of the olfactometer, and an air pump with a flow adjusted to 0.5 L/min was plugged to the opening at the top of the olfactometer. The time spent in each zone, odourised by the corresponding stimulus pipette and the number of transitions between the zones of the olfactometer, were observed during 15 min (i.e., one transition means an aphid moved from one zone to another). Olfactometers were changed, cleaned with detergent, and dried after each individual experiment and turned 90° in order to avoid any bias of orientation of the olfactometer within the setup. At least 30 insects were tested for each experimental condition (each aphid genotype/symbiotic status combination and each dose of EBF). 

After the experiment, the impact of EBF on aphid individuals was estimated by using a repulsivity score (hereafter noted RS) (see Equation (1)):

(1) $$\mathrm{RS}=\frac{\left(\mathrm{Cumulative}\;\mathrm{time}\;\mathrm{spent}\;\mathrm{in}\;\mathrm{the}\;\mathrm{two}\;\mathrm{EBF}\;\mathrm{zones}\;-\;\mathrm{Cumulative}\;\mathrm{time}\;\mathrm{spent}\;\mathrm{in}\;\mathrm{the}\;\mathrm{two}\;\mathrm{Hexane}\;\mathrm{zones}\right)}{\mathrm{Total}\;\mathrm{duration}\;\mathrm{of}\;\mathrm{the}\;\mathrm{experiment}}$$

A negative RS value indicates a repulsion of the aphid by the stimulus, while a positive RS value indicates an attraction of the aphid to the stimulus. Finally, a RS value close to 0 indicates no bias distribution of the aphid in the olfactometer device.

### 2.4. Electroantennogram

EAG recordings were done on L4 larvae of the three genotypes without facultative symbionts used in behavioural experiments and the *M. sativa* clone Ms2 injected with *H. defensa* originating from the same clone to test if behavioural sensitivity differences might originate from differences in the antennal sensitivity to EBF.

The insect was fixed on a holder with dental wax, and the antennae were kept in place with a bent insect pin. Electrodes were filled with Beadle Ephrussi Ringer and connected to an amplifier (axoclamp 2B, Molecular Devices, San Jose, CA, USA). The reference electrode was placed in the eye of the aphid, and the recording electrode was put in contact with the cut tip of one antenna. The signal was digitalised with an IDAC-4 device and recorded on EAG Pro software on a PC (Syntech, Kirchzarten, Germany). 

The antenna was superfused by a constant air stream (0.3 m/s) through a glass tube positioned at 10 mm from the aphid head and antennae. Two hundred microsecond odour pulses through the stimulation pipette were introduced into the constant air stream via a hole in the stimulation tube with a stimulation device (Stimulus controller CS 55, Syntech, Kirchzarten, Germany). A stimulation series always started with cis-3 hexenyl acetate, followed by hexane and increasing the EBF doses. At the end, cis-3 hexenyl acetate and hexane were tested again to evaluate a potential decline in responses. The interstimulus time interval was 60 s. To compensate for a decline in responses during the recording, amplitudes were normalised to cis-3 hexenyl acetate responses with the EAG Pro software. To compensate the differences in response to amplitudes, depending on each preparation, EAG responses to each dose of EBF were divided by the mean responses to hexane (before and after the EBF stimulation series).

### 2.5. Data Analyses

All statistical analyses were carried out using R version 3.6.2 [34].

#### 2.5.1. Olfactometry 

The first analysis consisted in testing whether the three aphid genotypes differed in their responses when exposed to EBF within the olfactometer. For this purpose, both the repulsivity score and the number of transitions of aphid individuals free of secondary symbionts within the olfactometer were tested against the aphid genotype, the EBF treatment, and the interaction between these two fixed factors. Then, we tested whether the symbiotic status of the aphid individuals influenced their responses to EBF exposure within the olfactometer. For this, both the repulsivity score and the number of transitions of the aphid individuals within the olfactometer were tested against their symbiotic status, the EBF treatment, and the interaction between these two fixed factors. Each pea aphid genotype was analysed separately in this second set of analyses. 

All repulsivity score data were analysed using a general linear model. Due to overdispersion in the dataset, all “number of transitions” data were analysed using a generalised linear model with a negative binomial error distribution and a log link function. In all models, the significance of the covariates was determined by using a likelihood ratio test. For each significant covariate, the associated estimated regression parameters were analysed to interpret the fitted models. Model assumptions were checked by plotting residuals versus fitted values and checking residual normality for general linear models.

#### 2.5.2. Electroantennogram

The first analysis consisted in testing whether the three aphid genotypes differed in their antennal sensitivity to EBF. For this purpose, the EAG responses of aphid individuals free of secondary symbionts were tested against the aphid genotype, the EBF dose, and the interaction between these two covariates. Then, we tested whether the symbiotic status of the aphid individuals influenced their antennal sensitivity to EBF. Considering the aphid genotype Ms2 only, the EAG responses of aphid individuals were tested against their symbiotic status (i.e., with or without *H. defensa*), the EBF treatment, and the interaction between these two covariates. To include the correlation between serial observations on the same aphid subject, the subject ID was implemented in the statistical models as a random effect. 

As decadic dilutions of EBF doses were applied, we log_10_-transformed this covariate before inclusion in the models. Additionally, before each statistical modelling, we checked the linearity assumption between the response and the EBF covariate. As we observed a linearity departure in all cases, we used generalised additive mixed models (GAMM) (*gamm* function of the *mgcv* package [35]). For each significant covariate, the associated estimated regression parameters were analysed to interpret the fitted models. Residuals of the fitted models were visually inspected (quantile–quantile plot and residuals vs. predicted values plot).

## 3. Results

### 3.1. Responses to EBF in the Olfactometer

Table 2 provides statistical details on the significance of the covariates included in the statistical models explaining aphid responses to EBF exposure within the olfactometer. 

#### 3.1.1. Effect of Aphid Genotypes on EBF Responses within the Olfactometer

**Figure 1:**
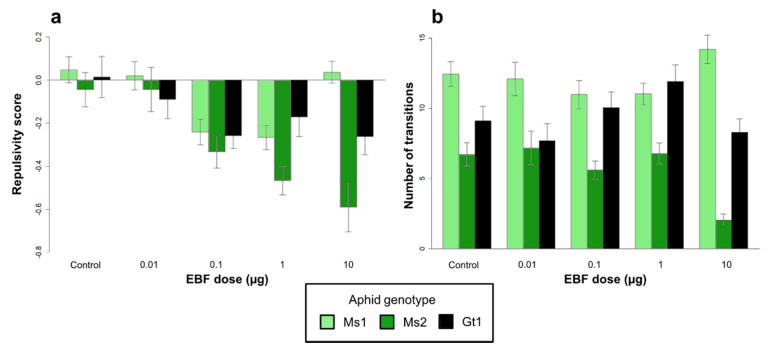
Repulsivity score (**a**) and number of transitions in olfactometer (**b**) at different E-β farnesene (EBF) doses for three *Acyrthosiphon pisum* genotypes free of secondary symbionts (i.e., Ms1, Ms2, and Gt1). Error bars show the standard error. N = 30 aphids for each stimulus and line.

EBF had increased repulsive effects with increased doses in the aposymbiotic aphids of all three tested genotypes (Figure 1a). However, this EBF dose effect varied according to the aphid genotype (significant interaction term, Table 2). For the Gt1 and Ms1 genotypes, the repulsivity decreased (Ms1) or remained unchanged (Gt1) at high doses compared to the Ms2 genotype (Figure 1a). EBF elicited a significantly stronger repulsive effect in the Ms2 genotype at the highest tested dose. Mobility, measured through transitions between different zones of the olfactometer, was significantly different between the three genotypes (Figure 1b). The Ms1 genotype was more mobile than the two other genotypes independently of the tested doses and even in the control situation without EBF (Figure 1b). However, this mobility variation between genotypes also depended on the EBF dose (significant interaction term, Table 2). The Ms2 genotype exhibited significantly less mobility at the highest tested EBF dose than the two other genotypes (Figure 1b). 

#### 3.1.2. Effect of Symbiotic Status on EBF Responses within the Olfactometer

**Figure 2:**
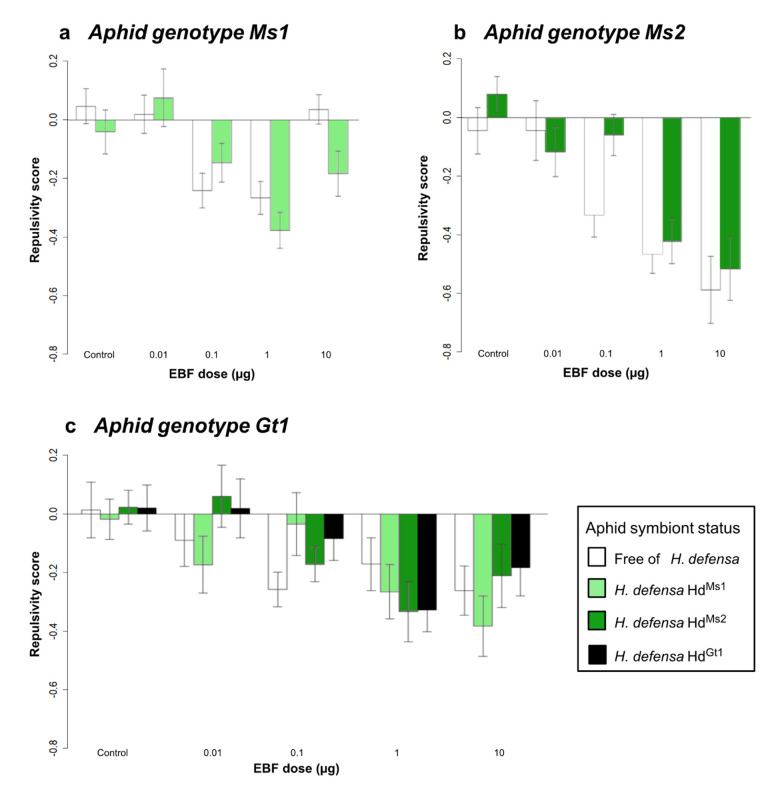
Repulsivity score at different EBF doses in *A. pisum* genotypes carrying or not carrying *Hamiltonella defensa.* White bars represent lines without *H. defensa*, and coloured bars represent lines with *H. defensa*. Different colours refer to different *H. defensa* strains. (**a**) Aphid genotype Ms1 (specialised on *Medicago sativa*). (**b**) Aphid genotype Ms2 (specialised on *M. sativa*). (**c**) Aphid genotype Gt1 (specialised on *Genista* *tinctoria*). Error bars show the standard error. N = 30 aphids for each stimulus and line.

In all three tested aphid genotypes, significant dose-dependent repulsive responses to EBF were observed, independently of the symbiotic status (Table 2 and Figure 2a–c). Although we found some differences in the repulsive scores, especially in Ms1 at 10 μg and Ms2 at 0.1 μg, they were not statistically supported, i.e., in all three genotypes, the presence of the original *H. defensa* did not significantly affect the repulsivity scores as compared to the corresponding aposymbiotic lines. Additionally, no significant effect of the origin of the injected *H. defensa* strain was observed when injected into the Gt1 genotype.

**Figure 3:**
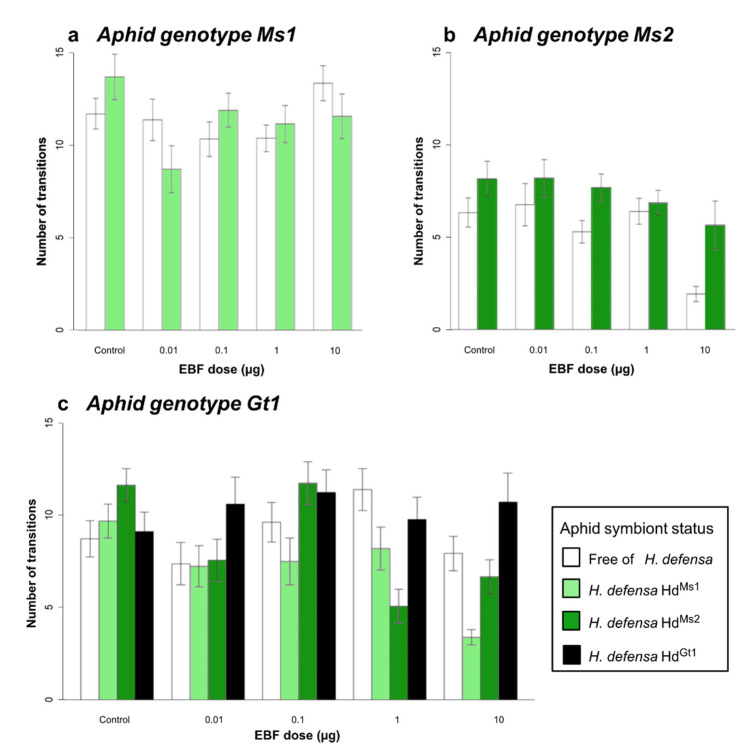
Number of transitions in the olfactometer at different EBF doses in *A. pisum* genotypes carrying or not carrying *H. defensa.* White bars represent lines without *H. defensa*, and coloured bars represent lines with *H. defensa*. Different colours refer to different *H. defensa* strains. (**a**) Aphid genotype Ms1 (specialised on *M. sativa*). (**b**) Aphid genotype Ms2 (specialised on *M. sativa*). (**c**) Aphid genotype Gt1 (specialised on *G. tinctoria*). Error bars show the standard error. N = 30 aphids for each stimulus and line.

In the Ms1 genotype, neither the EBF dose nor the symbiotic status influenced the mobility of aphid individuals (Table 2 and Figure 3a). In the Ms 2 genotype, the effect of infection with *H. defensa* on aphid mobility varied according to the EBF dose (significant interaction term, Table 2); while the EBF dose did not have any effect on infected aphids’ mobility, aposymbiotic individuals were less mobile at the highest tested dose (Figure 3b). Finally, in the Gt1 genotype, while the infection with the original *H. defensa* did not affect the mobility of aphids compared to aposymbiotic ones, injecting the *H. defensa* strain originating from the *M. sativa* biotype reduced the mobility significantly at high doses (significant interaction term, Table 2 and Figure 3c).

### 3.2. Effect of Genotype and Symbiotic Status on the Antennal Sensitivity to EBF

**Figure 4:**
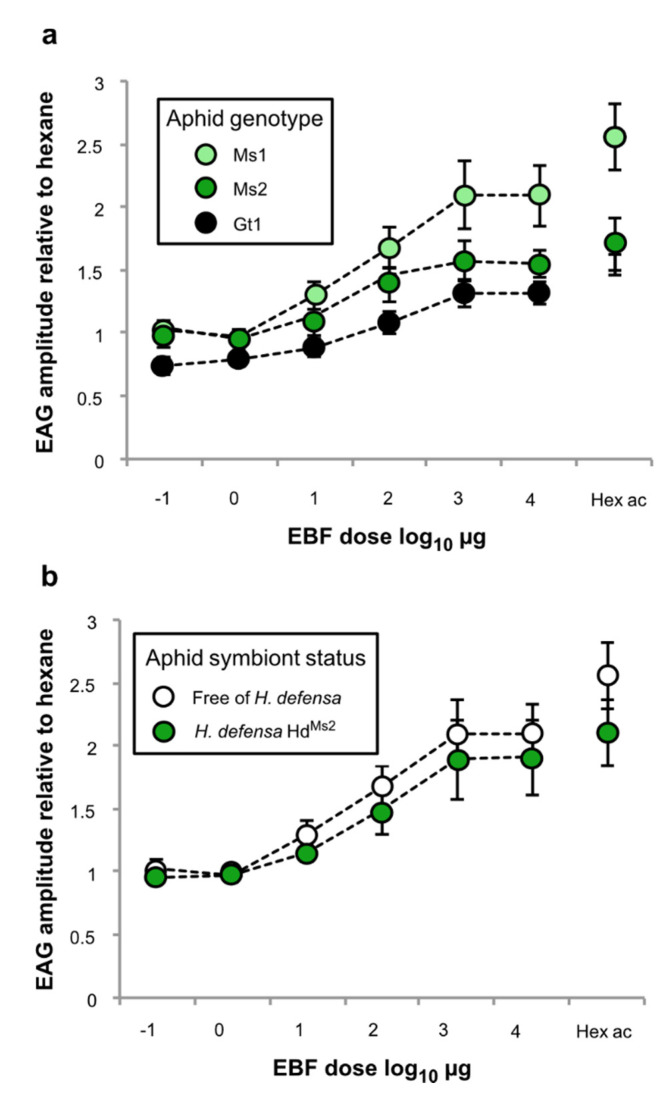
Relative electroantennogram (EAG) amplitudes in response to six EBF doses normalised with hexane responses for aposymbiotic individuals from the three *A. pisum* genotypes (**a**), and the Ms2 genotype with or without *H. defensa* (**b**). Error bars show the standard error. Detached points in the (**a**) and (**b**) graphs show normalised responses to cis-3-hexenyl acetate (a major volatile emitted by legume plants). EBF doses were log_10_-transformed for the analyses. N = 10 aphids for each stimulus and line.

Relative EAG responses to different doses of EBF revealed a clear nonlinear dose–response relationship in all tested clones (Figure 4a; GAMM: Ms1 genotype, F = 23.42, effective df (edf) = 3.16, *p* < 0.001; Ms2 genotype, F = 74.98, edf = 4.31, *p* < 0.001; and Gt1 genotype, F = 63.94, edf = 1.00, *p* < 0.001). The dose response curves of the three aposymbiotic lines differed significantly (GAMM: F = 9.77, df = 2, *p* < 0.001). Antennae of the M1 genotype responded with significantly larger relative EAG amplitudes to EBF than the other two genotypes (Figure 4a). Considering the Ms2 genotype only, we found that the dose response curves were, however, not significantly different between the aphid individuals infected or not with *H. defensa* (Figure 4b; GAMM: F = 2.97, df = 1, *p* = 0.087).

## 4. Discussion

Our results demonstrate that behavioural responses to the alarm pheromone EBF depend on the genotype of the pea aphid *A. pisum* and seem little influenced by the protective endosymbiont *H. defensa*. Electroantennographic responses to EBF correlated with the behavioural results, with differences in antennal sensitivity between biotypes and genotypes but not between lines carrying or not carrying *H. defensa*. 

EBF elicited increasing avoidance behaviour with increasing doses in all tested clones, correlated with increasing antennal response amplitudes. In some clones, however, the avoidance behaviour decreased at the highest dose, indicating a saturation effect.

Interestingly one of the two clones of the alfalfa biotype (Ms2) showed a stronger avoidance behaviour and lower locomotor activity than the second alfalfa-adapted genotype, whereas the dyer’s broom-specialised genotype showed the weakest avoidance behaviour and intermediate locomotor activity. Thus, the two evaluated variables seem not to be directly linked, even though a strong avoidance leads to a settlement in one of the control arms of the olfactometer and reduces the number of transitions between zones. Thus, high repulsive scores in a clone corresponded to low mobility at the corresponding EBF dose. Differences between the genotypes of the two different biotypes were not more important than differences between genotypes of the two clones of the same biotype.

These experiments, excluding environmental cues, provide similar results to earlier experiments evaluating different behavioural parameters (antennal movements, stylet withdrawal, etc.) on a plant when applying EBF in which differences between biotypes were already identified. However, differences between various genotypes of the same biotype were not analysed then [2]. Another behaviour, dropping from a host plant upon EBF exposure, did not reveal differences between, among others, biotypes adapted to alfalfa and dyer’s broom [15] (Ben-Ari et al., 2018); however, the Ms1 and Ms2 clones used in the present study were not investigated for this behaviour.

Our results demonstrate that both the behavioural avoidance and antennal detection of EBF are not uniform within a single biotype. However, behavioural and antennal responses were correlated in our study, which paves the way to investigate the underlying molecular mechanisms of differences between clones, such as the expression level of the EBF-binding ApisOR5 receptor. Indeed, previous studies found substantial genetic variations, as well as differential expressions, in olfactory genes between biotypes and genotypes of *A. pisum* [36,37,38].

The absence of significant effects of *H. defensa* on behavioural and antennal sensitivity to EBF in any of the tested aphid clones shows that this facultative endosymbiont seems not to influence the olfaction of aversive cues in *A. pisum.* Only the locomotor activity was significantly affected by the endosymbiont when the Ms2 and Gt1 clones were exposed to high doses of EBF. Earlier experiments already showed only minor and rather variable effects of endosymbionts on plant choice behaviours in different biotypes of the same aphid species; however, in this case, not only attractive olfactory cues but, also, visual and contact cues were available [14].

Contrary to our results, in a study in *Drosophila melanogaster*, gut microbiota influenced olfactory-guided foraging behaviours in the search for beneficial bacteria [25], but here, a more direct link can be expected than for bacteria present in other parts of the insect body. The absence of endosymbiont effects on the olfactory system in aphids shown here differs somewhat from the results found in two *Drosophila* species, where infection with the endosymbiont *Wolbachia pipientis* was shown to influence olfactory-guided orientation towards food cues [39]. In this latter study, *W. pipientis* effects on the responsiveness of the insect host to food cues and locomotor behaviour were, however, dependent on both the endosymbiont strain and the host species [39]. An improved response to food odours in *D. simulans*, when infected with *W. pipientis*, was also correlated with a higher expression of the odorant receptor gene *or83b* in the antenna [40]. The high variability of behavioural responses to EBF observed in our study confirms thus the importance of taking into account both bacterial and host strains when examining interactions of endosymbionts with insect sensory systems.

## 5. Conclusions

We show here that different *A. pisum* genotypes, both of the same and different biotypes, show different behavioural and antennal responses to the alarm pheromone EBF, whereas the facultative endosymbiont *H. defensa* had no significant effect on the repulsivity scores and antennal responses to the tested doses of EBF and only a minor influence on aphid mobility in the presence of high doses. We thus confirm here that olfactory-guided behaviour is more dependent on the aphid genotype than on the presence of endosymbiotic bacteria in *A. pisum*. However, further studies are needed to test more combinations between facultative endosymbionts and aphid genotypes or biotypes and to elucidate the mechanisms underlying the genetic variation in EBF responses.

## Figures and Tables

**Table 1:** Features of the eight lines of *Acyrthosiphon pisum* in the experiments.

Line Name	Aphid Genotype	Aphid Biotype	Secondary Symbiont Strain
Ms1-Hd^-^	Ms1	*Medicago sativa*	No secondary symbiont
Ms1-Hd^Ms1^			*Hamiltonella defensa* from Ms1
Ms2-Hd^-^	Ms2	*Medicago sativa*	No secondary symbiont
Ms2-Hd^Ms2^			*H. defensa* from Ms2
Gt1-Hd^-^	Gt1	*Genista tinctoria*	No secondary symbiont
Gt1-Hd^Gt1^			*H. defensa* from Gt1
Gt1-Hd ^Ms1^			*H. defensa* from Ms1
Gt1-Hd^Ms2^			*H. defensa* from Ms2

**Table 2:** Significance of the covariates included in the statistical models explaining both the repulsivity score and the number of transitions in the olfactometer at different E-β farnesene (EBF) doses in *Acyrthosiphon pisum* genotypes carrying or not carrying *Hamiltonella defensa.* Bold values highlight significant effects. Df: degrees of freedom.

		Variables					
		Repulsivity Score			Number of Transitions		
	Covariates	F	Df	*p*-Value	χ²	Df	*p*-Value
**Effect of aphid genotype**	Aphid genotype (1)	0.34	2	0.707	1345.37	2	**<0.001**
	EBF dose (2)	2.21	4	0.066	**9.82**	**4**	**0.043**
	(1) × (2)	**2.72**	8	**0.006**	**55.78**	**8**	**<0.001**
**Effect of aphid symbiotic status**	***Genotype Ms1***						
	Symbiotic status (1)	1.47	1	0.225	0.01	1	0.928
	EBF dose (2)	**9.64**	**4**	**<0.001**	8.35	4	0.079
	(1) × (2)	1.77	4	0.134	7.59	4	0.107
	***Genotype Ms2***						
	Symbiotic status (1)	2.63	1	0.105	2.05	1	0.152
	EBF dose (2)	**15.79**	**4**	**<0.001**	**45.62**	**4**	**<0.001**
	(1) × (2)	1.09	4	0.361	**15.58**	**4**	**0.003**
	***Genotype Gt1***						
	Symbiotic status (1)	0.50	3	0.681	2.87	3	0.411
	EBF dose (2)	**8.12**	**4**	**<0.001**	6.76	4	0.148
	(1) × (2)	0.98	12	0.464	**46.38**	**12**	**<0.001**

## Data Availability

Data are available as Supplementary Material (Data sheets EAG data and Behaviour data).

## Supplementary Materials

The following are available online at https://www.mdpi.com/2075-4450/12/1/43/s1: Figure S1. Four-way olfactometer used for behavioural tests. Data sheet EAG data. Data sheet Behaviour data.
