# Supplementary material for: Alarm Pheromone Responses Depend on Genotype, but Not on the Presence of Facultative Endosymbionts in the Pea Aphid Acyrthosiphon pisum
Source: Insects. 2021 Jan 8;12(1):43. doi: 10.3390/insects12010043 (PMC7826508; doi:10.3390/insects12010043)
Supplement: Supplementary file 1 [file insects-12-00043-s001.zip › supplementary material/Badji et al. Figure S1.docx]

**Alarm pheromone responses depend on genotype, but not on the presence of facultative endosymbionts in the pea aphid *Acyrthosiphon pisum***

Cesar Auguste Badji^1+^, Zoé Sol-Mochkovitch^1^, Charlotte Fallais^1^, Corentin Sochard ^2^, Jean-Christophe Simon ^3^ , Yannick Outreman ^2^ and Sylvia Anton ^1,^*

**Supplementary material**


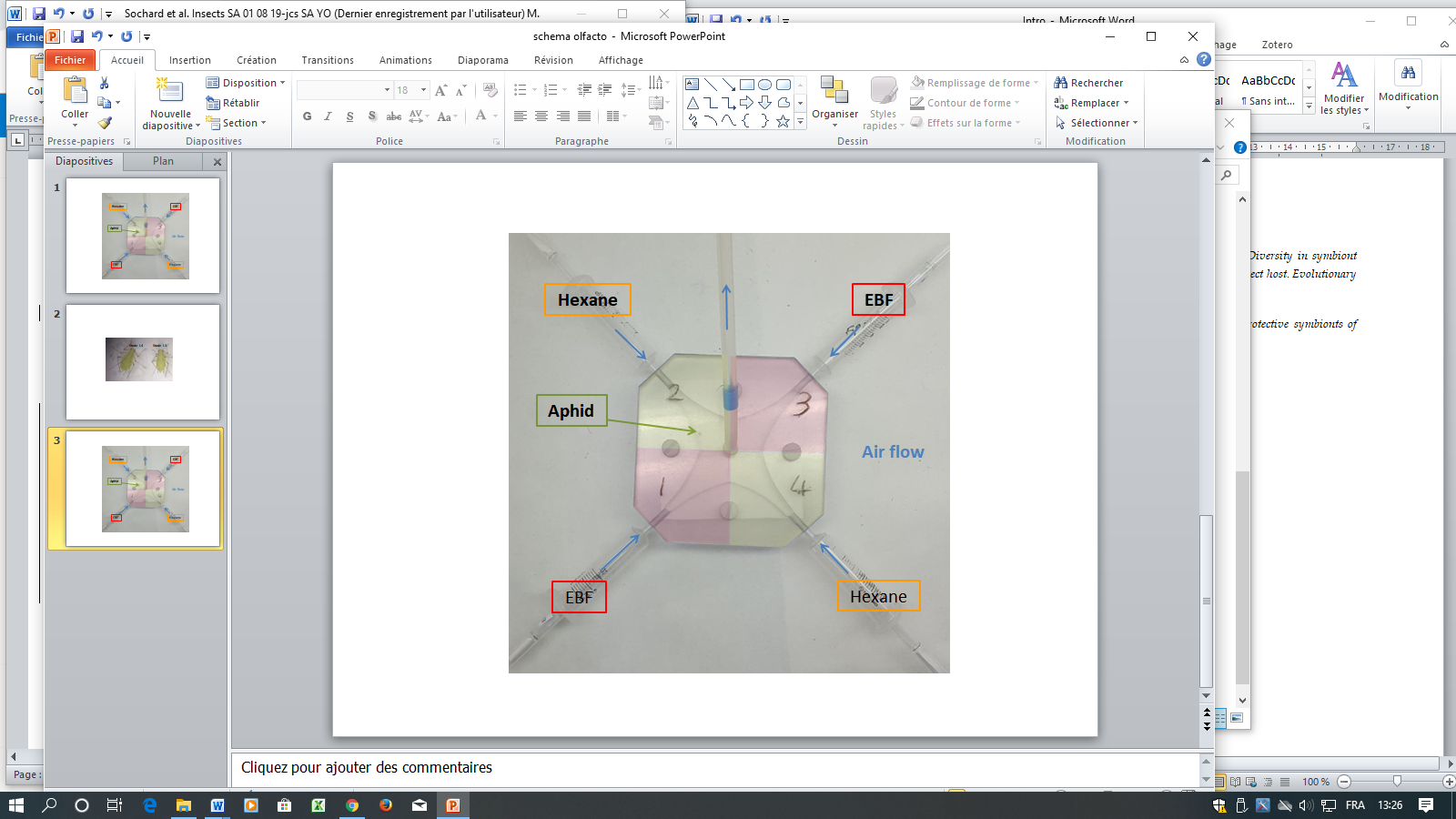


**Figure S1**. Four-way olfactometer used for behavioral tests.
